# Computation of Analytical Derivatives for Airborne TEM Inversion Using a Cole–Cole Parameterization Based on the Current Waveform of the Transmitter

**DOI:** 10.3390/s23010439

**Published:** 2022-12-31

**Authors:** Da Lei, Hao Ren, Changmin Fu, Zhongxing Wang, Qihui Zhen

**Affiliations:** 1 CAS Engineering Laboratory for Deep Resources Equipment and Technology, Institute of Geology and Geophysics, Chinese Academy of Sciences, Beijing 100029, China; 2 Innovation Academy for Earth Science, CAS, Beijing 100029, China; 3 College of Earth and Planetary Sciences, University of Chinese Academy of Sciences, Beijing 100049, China

**Keywords:** airborne transient electromagnetic, transmitter current waveform, analytical method, Cole–Cole model, induced polarization, inversion

## Abstract

Airborne transient electromagnetic (ATEM) technology is a technique often used in mineral exploration and geological mapping. Due to inductive polarization (IP) phenomena, the ATEM response curve often shows a negative response or declines rapidly to the attenuation curve. Traditional resistivity inversion techniques do not explain the IP response of a signal well, so the negative response is usually removed during data processing, resulting in a reduced correctness and authenticity of the findings. In this paper, in the parameter inversion based on the Cole–Cole model, the Jacobian matrix chain analysis method is used to calculate, and the current waveform calculation is also considered in the inversion. The results show that compared with the perturbation method, the analysis technique can greatly reduce the calculation time and improve the inversion efficiency. In the single-point one-dimensional inversion and lateral constraint quasi-two-dimensional inversion, the Cole–Cole four-parameter forward response has strong inversion accuracy, which can successfully invert the actual exploration content and the Cole–Cole four-parameter response. Some measured sounding data in the Qingchengzi survey area of Liaoning Province, China have a negative response to IP, and the resistivity scheme cannot be used alone for inversion, but the real underground resistivity structure can be obtained through the method studied in this paper, and good exploration results can be obtained.

## 1. Introduction

The ATEM method has the advantages of fast measurement and low cost, making it particularly suitable for deployment in inaccessible areas such as mountains, lakes, swamps, woods, and deserts. At present, this technology is mainly used for environmental engineering applications, groundwater and geothermal resource research, mineral exploration, and geological mapping [1,2,3,4,5,6,7]. Due to the continuous breakthrough of computer and digital electronic technology, the performance of the instrument, especially the signal-to-noise ratio (SNR) and acquisition time delay, has been significantly improved. Sign reversal is usually manifested in the measured ATEM response curve. Smith and Kelin [8] first demonstrated that sign reversal in the measurement response is caused by induced polarization (IP) effects. Inverting data with IP effects without considering IP parameters results in an erroneous resistivity model [9]. In the study of the IP effects of ATEM, Kozhevnikov and Antonov [10,11] used numerical simulations to study the ground transient electromagnetic inversion of the uniform polarization half-space model and two-layer model. Kratzer and Macnae [12] devised an approximate interpretation method that decomposes the observed data into an EM inductive response and an excitation polarization response. In the study of ATEM inversion, Viezzoli et al. [6] successfully inverted zero-frequency resistivity and chargeability from ATEM data under specific model constraints. Kang and Oldenburg [13] proposed a 3D inversion technique for ATEM data. Many researchers have conducted in-depth studies on the IP effect of ATEM and have had success with actual exploration [9,14].

The existing AEROTEM, Hoistem, VTEM, HeliGEOTEM, Geotech, and SkyTEM transmitters have their own different current waveforms. Christiansen et al. [15] found that different transmitter current waveforms have a great influence on the observations. The simulation of transmitter current waveforms was also considered in this forward modeling [6,13,16,17]. Kang and Oldenburg [13] also investigated the calculation of transmitter current waveforms in the inversion.

In summary, the IP effect and transmitter waveform of ATEM have a great influence on the inversion of ATEM data, and the forward and the inversion with the transmitter waveform require a long calculation time. In order to improve the calculation efficiency, the transmitter current waveform is taken into consideration, and the Jacobi matrix chain analysis method based on the Cole–Cole model is studied. This method inverts the measured data to obtain the true underground resistivity structure, greatly reduces calculation time, and improves inversion efficiency, leading to successful exploration outcomes.

## 2. The Basic Principles of Forward and Inversion

In 1D model forward modeling, the device system is the center loop source, the *z*-axis is positive downwards, the *x* and *y* axes are on the horizontal plane, and the center of the loop is directly above the rectangle coordinate system’s origin. In the quasi-static case, the vertical component of the frequency domain magnetic field strength at the center of the torus is expressed as [18].

(1) $$H_{z}=\frac{Ia}{2}\int_{0}^{\infty }\left[e^{-\lambda \left(z+hg\right)}+r_{TE}e^{\lambda \left(z-hg\right)}\right]\lambda J_{1}\left(\lambda a\right)d\lambda .$$

where *hg* is the height of the transmitter coil from ground (*hg* = −*z* ≥ 0), *I* is the amplitude of the transmit current, *a* is the radius of the transmitter coil, *z* is the vertical height of the receiving coil, *J*_1_(λ*a*) is the first-order Bessel function, λ is the integral variable, and $r_{TE}$ is reflection coefficient.

The frequency domain response was calculated with 47 Hankel filter coefficients proposed by Guptasarma et al. [19]. For calculating the time domain response, Wang [20] proposed 250 sinusoidal numerical filter coefficients. The time domain response expression is:

(2) $$\frac{dh_{z}\left(t\right)}{dt}=-\frac{1}{t}\sqrt{\frac{2}{\pi }}\sum_{n=-\infty }^{+\infty }Im\left[H_{z}(\frac{e^{n\Delta }}{t})\right]\cdot w_{n}.$$

where Δ is the sampling interval, *n* is the number of filtering coefficients, $H_{z}\;$ is the frequency domain response function, and *w_n_* is the filtering coefficient.

The Cole–Cole composite resistivity model [21] replaces the mathematical formula for the impact of resistivity in forward mode, the IP effects of rocks and ores.

(3) $$\rho \left(i\omega \right)=\rho_{0}\left(1-m\left(1-\frac{1}{1+{\left(i\omega \tau \right)}^{c}}\right)\right).$$

where *τ* is the relaxation time (or time constant) (s or ms), ρ_0_ is the zero-frequency resistivity (Ω·m), *m* is the chargeability (V/V), *ω* is the angular frequency (radian), and *c* is the frequency exponent. According to [21], Table 1 shows the statistical results of in situ conductivity spectrum measurements on typical charged mineralization events. The parameters of the Cole–Cole model often fall into the following ranges: ρ_0_ = 10^−2^–10^4^ (Ω·m), *m* = 0.1–0.98 (V/V), *τ* = 10^−5^–10^5^ (s), and *c* = 0.1–0.6.

### 2.1. Transmitting Waveform Response

Convolutional calculations can be used to determine the response of various emitted waveforms, enabling one to derive the electromagnetic response of any emitted waveform in the time domain. In practical applications, airborne electromagnetic transmitter systems often use different transmitter waveforms to acquire data. Using the convolution theorem and the relationship between step response and impulse response, the convolution formula of electromagnetic response in the time domain of arbitrary emission current is obtained:

(4) $$\frac{dH_{z}\left(t\right)}{dt}=\frac{dI\left(t\right)}{dt}*h_{zi}\left(t\right)=-\frac{dI\left(t\right)}{dt}*\frac{dh_{z}\left(t\right)}{dt}=-\frac{d^{2}I\left(t\right)}{dt^{2}}*h_{z}\left(t\right).$$

where *I*(*t*) is the emission current, and $h_{zi}\left(t\right)$ and $h_{z}\left(t\right)$ are the impulse and step responses of the airborne electromagnetic system, respectively. It is important to note that the step response and impulse response of any system have an integral/differential relationship.

**Figure 1:**
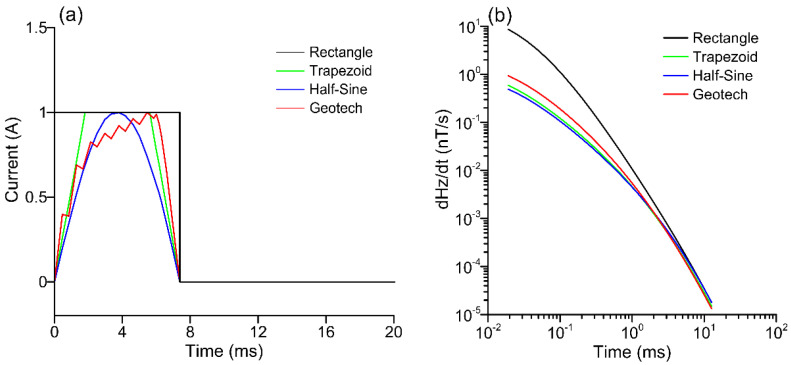
Comparison of four different types of transmitted current signals and induced electromotive force curves for center loops. (**a**) Geotech instrument waveform, rectangular, trapezoidal, and semi-sinusoidal. (**b**) One-dimensional response of a uniform semi-spatial model of four waveforms. The start time in (**b**) corresponds to the pulse width of the four emitted waves in (**a**), which is 7.385 ms.

Taking the helicopter-borne with center loop device as an example, the time derivative of the magnetic induction intensity ∂*H*_z_(*t*)/∂*t* under the excitation of four different transmitter waveforms is compared. The following parameters are provided for the model: The radius of the transmitter coil is 15 m, the height from the ground is 50 m, the semi-spatial resistivity is uniformly 100 Ω·m, and the transmitter current is 100 amperes. The time for the rising and falling edges of the rectangle is 0 ms, while the single pulse power supply time for the four waveforms is 7.385 ms. The ascending and falling edges of the trapezoid have a time of 1.71 ms, the power-supply and power-off times of the half-sine wave are 3.61 ms, the power-supply and power-off times of the Geotech instrument waveforms are 6.355 ms and 1.03 ms, respectively, and the waveforms of the four transmitter current pulse signals are shown in Figure 1a.

The 1-D response curves of four waveforms to a half-space model are shown in Figure 1b. The half-sine wave produces the least electromagnetic intensity, while the rectangular wave produces the largest electromagnetic response, followed by the Geotech instrument waveform. The four results are essentially the same in the late stages, and the transient responses of the four waveforms differ greatly in the early stages. Geotech transmitter waveforms are used for all simulations and inversions in the final part of this study. The calculations produced in this study were compared with those of the Yin et al. [17] method.

**Figure 2:**
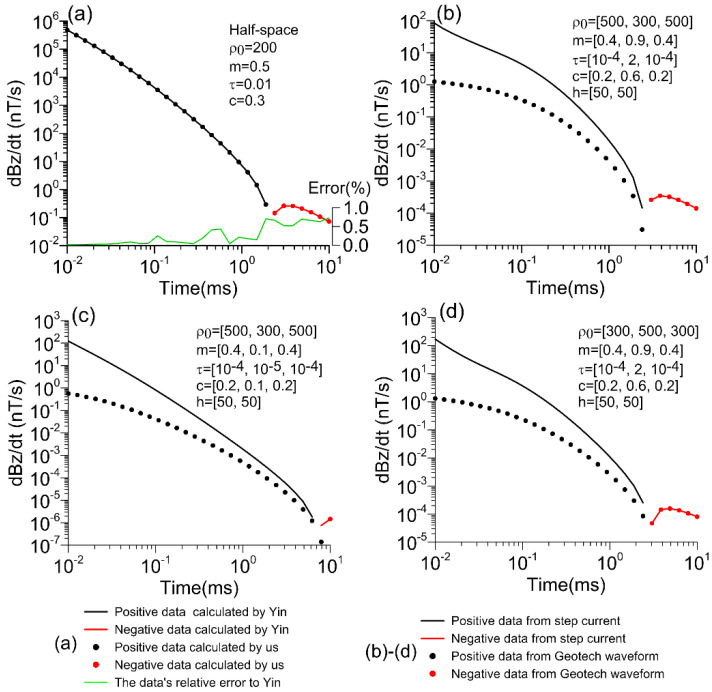
Verification of the accuracy of half-space and three-layer models. (**a**) Waveform of step current; Yin et al. [17] simulated the data (lines) and the data calculated in this study (circle markers). Although there are slight differences between the two methods, they are not visible in the diagram. The relative error of the data calculated in this study relative to the Yin data is represented by a green line. (**b**–**d**) Data calculated from Geotech waveforms (circle markers) and step currents (lines), respectively; black (positive) and red (negative).

To confirm the accuracy of a one-dimensional orthography using a step waveform, the parameters for the uniformly polarized half-space model are set to ρ_0_ = 200 Ω·m, *m* = 0.5, *τ* = 0.01 s, and *c* = 0.3. The transmitter has a height of 30 m, current of 100 amps, and a coil radius of 15 m. The numerical solution of the forward program is highly consistent with the algorithmic results of Yin et al. [17]. As shown in Figure 2a (the dotted line in the image indicates a negative reaction), this verifies the correctness of the program. In addition, three three-layer models were designed for Geotech waveform verification and one-dimensional orthography. The first model MD-1 is ρ_0_ = {500, 300, 500} Ω·m, *m* = {0.4, 0.9, 0.4}, *τ* = {10^−4^, 2, 10^−4^} s, *c* = {0.2, 0.6, 0.2}, *h* = {50, 50} m, *h* denotes the layer thickness dataset in this study; the second model MD-2 is ρ_0_ = {500, 300, 500} Ω·m, *m* = {0.4, 0.1, 0.4}, *τ* = {10^−4^, 10^−5^, 10^−4^} s, *c* = {0.2, 0.1, 0.2}, *h* = {50, 50} m; the third model MD-3 is ρ_0_ = {300, 500, 300} Ω·m, *m* = {0.4, 0.9, 0.4}, *τ* = {10^−4^, 2, 10^−4^} s, *c* = {0.2, 0.6, 0.2}, *h* = {50, 50} m; the simulation results of the three models for the step waveform and the Geotech waveform are shown in Figure 2b–d, respectively. Figure 2b–d shows step waveform response values that are significantly larger than the results of the Geotech waveform in the early stages. The responses of the high-chargeability, low-resistivity layer model (MD-1) and high-resistivity layer model (MD-3) have significant negative anomalies in the late stages (Figure 2b,d), while the low-chargeability model MD-2 has weak negative anomalies. This verifies the correctness of the program’s simulation of the transmitter waveform of the Cole–Cole model.

### 2.2. Analytical Formulae

In inversion, each iteration must compute the Jacobi matrix, and the computational time is mainly spent on finding the Jacobi matrix. In this paper, the analytical formula for obtaining the Jacobi matrix in airborne transient electromagnetism considering the excitation effect is derived. Suppose the model has an N-layer, and the bias derivative of the time domain electromagnetic response of the transmitter current of any waveform is as follows:

(5) $$\frac{\partial }{\partial p}\frac{dH_{z}\left(t\right)}{dt}=-\frac{dI\left(t\right)}{dt}*\frac{\partial }{\partial p}\frac{dh_{z}\left(t\right)}{dt}=-\frac{d^{2}I\left(t\right)}{dt^{2}}*\frac{\partial }{\partial p}H_{z}\left(t\right).$$

where *p* represents model parameter set [ρ_01_, …, ρ_0N_; *m*_1,_ …, *m*_N_; *τ*_1,_ …, *τ*_N_; *c*_1,_ …, *c*_N_; h_1,_ …, h_N−1_]. According to Equation (1), the kernel function is *K*:

(6) $$K=\frac{Ia\lambda }{2}\left(e^{-\lambda \left(z+hg\right)}+r_{TE}e^{\lambda \left(z-hg\right)}\right).$$

(7) $$r_{TE}=\frac{\lambda -\hat{\mu_{1}}}{\lambda +\hat{\mu_{1}}}.$$

(8) $${\hat{\mu }}_{1}=\mu_{1}\frac{{\hat{\mu }}_{2}+\mu_{1}\tan h\left(\mu_{1}h_{1}\right)}{\mu_{1}+{\hat{\mu }}_{2}\tan h\left(\mu_{1}h_{1}\right)}.$$

(9) $${\hat{\mu }}_{j}=\mu_{j}\frac{{\hat{\mu }}_{j+1}+\mu_{j}\tan h\left(\mu_{j}h_{j}\right)}{\mu_{j}+{\hat{\mu }}_{j+1}\tan h\left(\mu_{j}h_{j}\right)}.$$

(10) $${\hat{\mu }}_{N}=\mu_{N}.$$

(11) $$\mu_{j}={\left(\lambda^{2}-k_{j}^{2}\right)}^{1/2}.$$

(12) $$k_{j}^{2}=-i\omega \mu_{j}/\rho_{j}.$$

where *ω* is the angular frequency, *k_j_* is the wave number of the *j* layer, h*_j_* is the thickness of the *j* layer, ρ*_j_* is the resistivity of the *j* layer, and *μ_j_* is the permeability of the *j* layer. It is generally considered that the large geomagnetic permeability *μ_j_* is equal to the permeability of *μ*_0_ in a vacuum.

Derivative of chain rules for IP parameters and depth.

(13) $$\frac{\partial K}{\partial \rho_{0}{}_{j}}=\frac{\partial K}{\partial {\hat{\mu }}_{1}}\frac{\partial {\hat{\mu }}_{1}}{\partial {\hat{\mu }}_{2}}\frac{\partial {\hat{\mu }}_{2}}{\partial {\hat{\mu }}_{3}}\cdots \frac{\partial {\hat{\mu }}_{j}}{\partial \mu_{j}}\frac{\partial \mu_{j}}{\partial \rho_{j}}\frac{\partial \rho_{j}}{\partial \rho_{0}{}_{j}}.$$

(14) $$\frac{\partial K}{\partial m_{j}}=\frac{\partial K}{\partial {\hat{\mu }}_{1}}\frac{\partial {\hat{\mu }}_{1}}{\partial {\hat{\mu }}_{2}}\frac{\partial {\hat{\mu }}_{2}}{\partial {\hat{\mu }}_{3}}\cdots \frac{\partial {\hat{\mu }}_{j}}{\partial \mu_{j}}\frac{\partial \mu_{j}}{\partial \rho_{j}}\frac{\partial \rho_{j}}{\partial m_{j}}.$$

(15) $$\frac{\partial K}{\partial \tau_{j}}=\frac{\partial K}{\partial {\hat{\mu }}_{1}}\frac{\partial {\hat{\mu }}_{1}}{\partial {\hat{\mu }}_{2}}\frac{\partial {\hat{\mu }}_{2}}{\partial {\hat{\mu }}_{3}}\cdots \frac{\partial {\hat{\mu }}_{j}}{\partial \mu_{j}}\frac{\partial \mu_{j}}{\partial \rho_{j}}\frac{\partial \rho_{j}}{\partial \tau_{j}}.$$

(16) $$\frac{\partial K}{\partial c_{j}}=\frac{\partial K}{\partial {\hat{\mu }}_{1}}\frac{\partial {\hat{\mu }}_{1}}{\partial {\hat{\mu }}_{2}}\frac{\partial {\hat{\mu }}_{2}}{\partial {\hat{\mu }}_{3}}\cdots \frac{\partial {\hat{\mu }}_{j}}{\partial \mu_{j}}\frac{\partial \mu_{j}}{\partial \rho_{j}}\frac{\partial \rho_{j}}{\partial c_{j}}.$$

(17) $$\frac{\partial K}{\partial h_{j}}=\frac{\partial K}{\partial {\hat{\mu }}_{1}}\frac{\partial {\hat{\mu }}_{1}}{\partial {\hat{\mu }}_{2}}\frac{\partial {\hat{\mu }}_{2}}{\partial {\hat{\mu }}_{3}}\cdots \frac{\partial {\hat{\mu }}_{j}}{\partial \mu_{j}}\frac{\partial \mu_{j}}{\partial d_{j}}.$$

(18) $$\frac{\partial K}{\partial {\hat{\mu }}_{1}}=\frac{-Ia\lambda^{2}}{{\left(\lambda +{\hat{\mu }}_{1}\right)}^{2}}e^{\lambda \left(z-hg\right)}.$$

(19) $$\frac{\partial {\hat{\mu }}_{j}}{\partial {\hat{\mu }}_{j+1}}=\mu_{j}^{2}\left[1-\tan h^{2}\left(\mu_{j}h_{j}\right)\right]/{\left[\mu_{j}+{\hat{\mu }}_{j+1}\tan h\left(\mu_{j}h_{j}\right)\right]}^{2}.$$

(20) $$\frac{\partial {\hat{\mu }}_{j}}{\partial \mu_{j}}=A/{\left[\mu_{j}+{\hat{\mu }}_{j+1}\tan h\left(\mu_{j}h_{j}\right)\right]}^{2}.$$

(21) $$A=-\mu_{j}h_{j}{\hat{\mu }}_{j+1}^{2}+\mu_{j}^{3}h_{j}+\left(\mu_{j}^{2}+{\hat{\mu }}_{j}^{2}\right)\tan h\left(\mu_{j}h_{j}\right)+\;\left(\mu_{j}h_{j}{\hat{\mu }}_{j+1}^{2}-\mu_{j}^{3}h_{j}+2\mu_{j}{\hat{\mu }}_{j}\right)\tan h^{2}\left(\mu_{j}h_{j}\right).$$

(22) $$\frac{\partial {\hat{\mu }}_{N}}{\partial \mu_{N}}=1.$$

(23) $$\frac{\partial \mu_{j}}{\partial \rho_{j}}=\frac{-i\omega \mu }{2\mu_{j}\rho_{j}^{2}}.$$

The derivative of ρ with respect to the IP parameter can be obtained from Equation (3).

(24) $$\frac{\partial \rho_{j}}{\partial \rho_{0}{}_{j}}=\left(1-m_{j}\left(1-\frac{1}{1+{\left(i\omega \tau_{j}\right)}^{c_{j}}}\right)\right).$$

(25) $$\frac{\partial \rho_{j}}{\partial m_{j}}=-\rho_{0}{}_{j}\left(1-\frac{1}{1+{\left(i\omega \tau_{j}\right)}^{c_{j}}}\right).$$

(26) $$\frac{\partial \rho_{j}}{\partial \tau_{j}}=-\rho_{0}{}_{j}m_{j}c_{j}i\omega {\left(i\omega \tau_{j}\right)}^{c_{j}-1}/{\left(1+{\left(i\omega \tau_{j}\right)}^{c_{j}}\right)}^{2}.$$

(27) $$\frac{\partial \rho_{j}}{\partial c_{j}}=-\rho_{0}{}_{j}m_{j}{\left(i\omega \tau_{j}\right)}^{c_{j}}ln\left(i\omega \tau_{j}\right)/{\left(1+{\left(i\omega \tau_{j}\right)}^{c_{j}}\right)}^{2}.$$

The derivative of the thickness can be obtained from Equation (11):

(28) $$\frac{\partial {\hat{\mu }}_{j}}{\partial h_{j}}=\mu_{j}^{2}\left(\mu_{j}^{2}-{\hat{\mu }}_{j+1}^{2}\right)\left[1-\tan h^{2}\left(\mu_{j}h_{j}\right)\right]/{\left[\mu_{j}+{\hat{\mu }}_{j+1}\tan h\left(\mu_{j}h_{j}\right)\right]}^{2}.$$

When we take the derivatives of $\partial K/\partial \rho_{0}{}_{j}$, $\partial K/\partial m_{j}$,$\;\partial K/\partial \tau_{j}$, $\partial K/\partial c_{j}$, $\partial K/\partial h_{j}$, we only need to take the intermediate values of $\;\partial K/\partial \rho_{0}{}_{j+1},\;\partial K/\partial m_{j+1},\;\partial K/\partial \tau_{j+1},$ $\partial K/\partial c_{j+1},\;\;\partial K/\partial h_{j+1}.$ Therefore, the derivatives of the last layer are calculated$,\partial K/\partial \rho_{0}{}_{N}$, $\partial K/\partial m_{N}$,$\;\partial K/\partial \tau_{N}$, $\partial K/\partial c_{N}$, and the derivatives of the remaining layer can be used directly from the intermediate calculated values. Finally, $\partial K/\partial \rho_{0}$, ∂K/∂m, ∂K/∂τ , and ∂K/∂h are placed in a matrix, and in the time domain the Jacobian matrix is obtained by Hankel transformation and sinusoidal transformation, and the Jacobian matrix of the transmitting waveform is obtained by convolution of the transmitted waveform in the time domain.

### 2.3. Contrast of Analytical and Perturbation Methods

**Figure 3:**
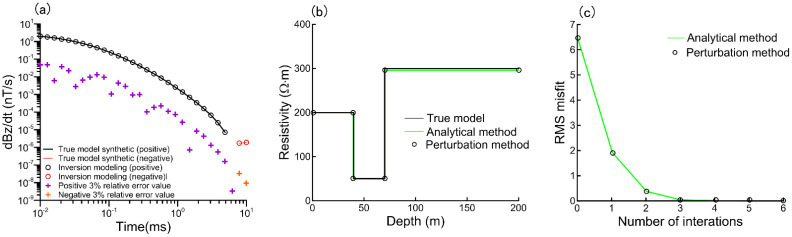
Model inversion. (**a**) Data fitting between simulated data plus 3% relative error normally distributed noise levels and inversion model modeling, line for forward responses and circles for the synthetic data; Black (positive) and red (negative); 3% relative error values (crossover), purple (positive), and orange (negative). (**b**) Inversion results of the analytical and perturbation methods. (**c**) Data RMS misfit.

The perturbation method is commonly used to compute the Jacobi matrix. The perturbation approach first applies a minor disturbance to a single independent variable before replacing the Jacobian matrix with a differential calculation based on the electromagnetic response acquired from forward modeling. To ensure dependability, we compare the analytical solution to the perturbation approach. Using the fixed *m*, *τ*, and *c*, we invert the two approaches. The three-layer model consists of the first layer with (ρ_01_ = 200 Ω·m, *m*_1_ = 0.2, *τ*_1_ = 0.001 s, *c*_1_ = 0.3, *h*_1_ = 40 m), the second layer with (ρ_02_ = 50 Ω·m, *m*_2_ = 0.2, *τ*_2_ = 0.001 s, *c*_2_ = 0.3, *h*_2_ = 30 m), and the basal layer with (ρ_03_ = 300 Ω·m, *m*_3_ = 0.2, *τ*_3_ = 0.001 s, *c*_3_ = 0.3). Three subsurface layers are predetermined to exist. Each layer is 50 m thick. The starting model is a homogeneous half-space of 100 Ω·m. The resistivity constraint range is 1 to 5000 Ω·m, with an anticipated misfit of 0.01. The transmitter coil radius in the center loop arrangement is 15 m, the Geotech waveform is used for transmission, the transmitter current is 100 A, and the transmitter height is 30 m. The simulated data is given a relative error normal distribution noise level of 3% for the inversion. Figure 3a displays the noise with a normal distribution.

The root mean square (RMS) error criteria was used to assess the degree of fit. The error function is represented by the calculation.

(29) $$\mathrm{RMS}=\sqrt{\sum_{i=1}^{NK}{\left(\frac{H_{obs}{}_{i}-H_{pre}{}_{i}}{H_{obs}{}_{i}}\right)}^{2}/NK}$$

where the number of datasets is *NK*, the observed or synthetic data is $H_{obs}$, and the predictive datasets is $H_{pre}$.

The inversion result is shown in Figure 3, showing that the final fit difference between the two inversion methods is basically the same for the same model and iteration time. The relative error of the corresponding Jacobian matrix elements calculated by the two methods is less than 10^−5^, and this analytical method can accurately meet the Jacobian matrix calculation requirements.

The accuracy of Cole–Cole parameter parsing methods is verified on a three-layer model. The three-layer model consists of the first layer (ρ_01_ = 100 Ω·m, *m*_1_ = 0.1, *τ*_1_ = 0.001 s, *c*_1_ = 0.3, *h*_1_ = 100 m), the second layer (ρ_02_ = 40 Ω·m, *m*_2_ = 0.5, *τ*_2_ = 0.001 s, *c*_2_ = 0.3, *h*_2_ = 30 m), and the base layer (ρ_03_ = 500 Ω·m, *m*_3_ = 0.2, *τ*_3_ = 0.001 s, *c*_3_ = 0.3). Table 2 shows the perturbation method (the amount of disturbance is 1% of each parameter) and Jacobian analytical method values for the Cole–Cole parameters calculated with the three-layer model parameters, as well as the relative error values of the perturbation method and Jacobian matrix value. The results of the two methods are very similar, and the relative error between the Jacobian analytical method and the perturbation method is less than 1.65%, demonstrating the accuracy and reliability of the Jacobian matrix. The analytical method takes 46.33 s to calculate, whereas the perturbation method takes 2,380.56 s, demonstrating that the analytical method is much faster to calculate than the perturbation method.

### 2.4. Sensitivity Analysis

To analyze the sensitivity of 1-D inversion of the ATEM response to the parameters of the Cole–Cole model in the time domain, the sensitivity indicator *Sen* of the Jacobian matrix can be used to indirectly reflect the sensitivity of different parameters to positive calculus at different times.

(30) $$Sen=\left|\frac{\partial }{\partial p}\frac{dH_{z}(t)}{dt}\right|.$$

where *Sen* represents the change in the forward operator caused by the unit perturbation of the model parameters. *Sen* can be obtained from the absolute value of the Jacobian determinant in the Equations (13)–(28) and (30). Obviously, the larger the *Sen* value, the more sensitive the forward operator is to changes in the input model. As a result, the sensitivity analysis of the model parameters can be calculated. This is critical for the resolution assessment of the input model and the integrity assessment of the model.

**Figure 4:**
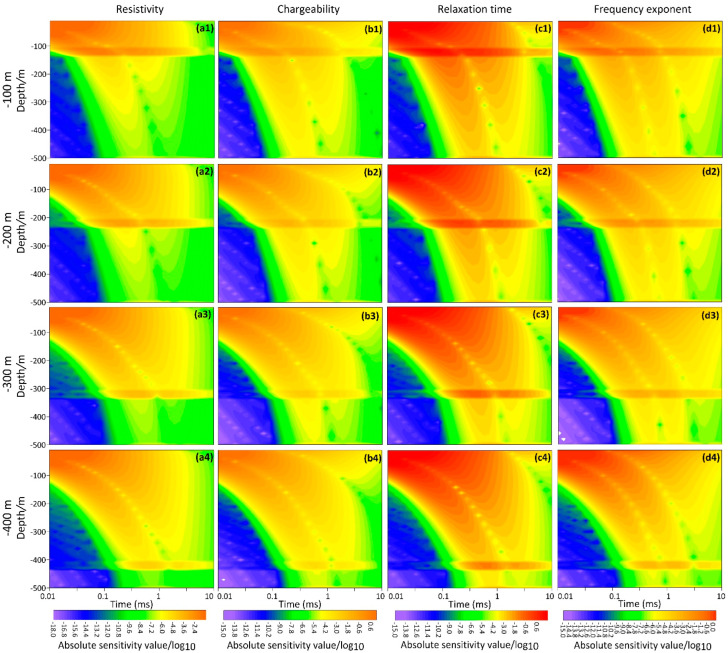
Absolute values of the sensitivity for the four models through the Jacobian matrix. Four depths of a resistive layer, each 30 m thick in the three-layer model. (**a1**–**d1**) 100 m, (**a2**–**d2**) 200 m, (**a3**–**d3**) 300 m, and (**a4**–**d4**) 400 m for three layers with a resistive basement and a conductive overburden are modelled. The (**a1**–**a4**) resistivity, (**b1**–**b4**) chargeability, (**c1**–**c4**) relaxation time, and (**d1**–**d4**) frequency exponent are shown for each model.

The sensitivity response of the low-resistivity layers at different depths is shown in Figure 4. The low-resistivity layer of the model is the second layer, which is moved down from 100 m to a depth of 200 m, 300 m, or 400 m, and the absolute sensitivity is calculated by Jacobian analysis. The absolute sensitivity of the four Cole–Cole parameters shows a well-layered shape when the low-resistivity layer is at a depth of 100 m (Figure 4a1–d1), and there are high anomalies at 100 m. The high anomaly moves down to these three depths as the low-resistivity layer moves down to 200 m, 300 m, and 400 m, indicating that the sensitivity anomaly of the low-resistivity layer gradually shifts from early to late time. It shows that the absolute sensitivity of the four depths has a good sensitivity abnormality at the corresponding depth, and the method has a better resolution for the four parameters of Cole–Cole in the low-resistivity layer.

### 2.5. Synthetic Inversion

#### 2.5.1. Inversion of a Single Sounding Point

The simulation results of the following two models are used to check the inversion of the damping least squares technique of the Jacobian matrix based on the chain rule. The transmitter has a height of 50 m, an electric current of 100 amps, and a coil radius of 15 m. Model I consists of the first layer (ρ_01_ = 200 Ω·m, *m*_1_ = 0.06, *τ*_1_ = 0.02 s, *c*_1_ = 0.2, *h*_1_ = 100 m), the second layer (ρ_02_ = 50 Ω·m, *m*_2_ = 0.7, *τ*_2_ = 0.01 s, *c*_2_ = 0.5, *h*_2_ = 100 m), and the base layer (ρ_03_ = 500 Ω·m, *m*_3_ = 0.06, *τ*_3_ = 0.02 s, *c*_3_ = 0.03). Model II consists of the first layer (ρ_01_ = 100 Ω·m, *m*_1_ = 0.1, *τ*_1_ = 0.001 s, *c*_1_ = 0.1, *h*_1_ = 100 m), the second layer (ρ_02_ = 500 Ω·m, *m*_2_ = 0.6, *τ*_2_ = 0.006 s, *c*_2_ = 0.6, *h*_2_ = 100 m), and the base layer (ρ_03_ = 100 Ω·m, *m*_3_ = 0.1, *τ*_3_ = 0.001 s, *c*_3_ = 0.1). During the data inversion process simulated by these two models, the initial inversion model has four layers; each layer is the same (ρ_0_ = 300 Ω·m, *m* = 0.3, *τ* = 0.2 s, *c* = 0.4, *h* = 80 m).

**Figure 5:**
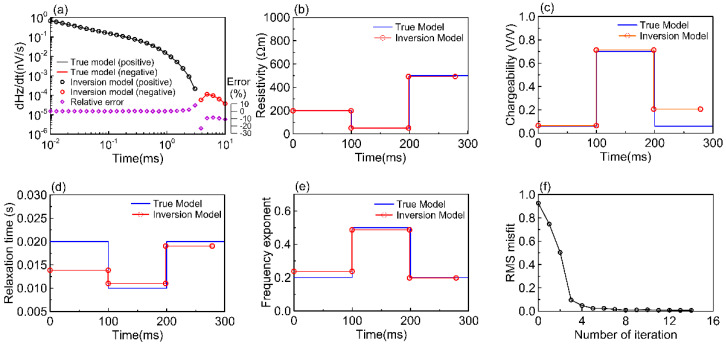
Model I inversion results of the Geotech waveform. (**a**) Data RMS misfit between true model modeling and inversion model simulation. Synthesized data (line) and forward response (circle mark); black (positive) and red (negative); relative error (purple diamond). (**b**–**e**) Represent resistivity, chargeability, relaxation time, frequency exponent, in that order. (**f**) RMS misfit for the number of iterations.

**Figure 6:**
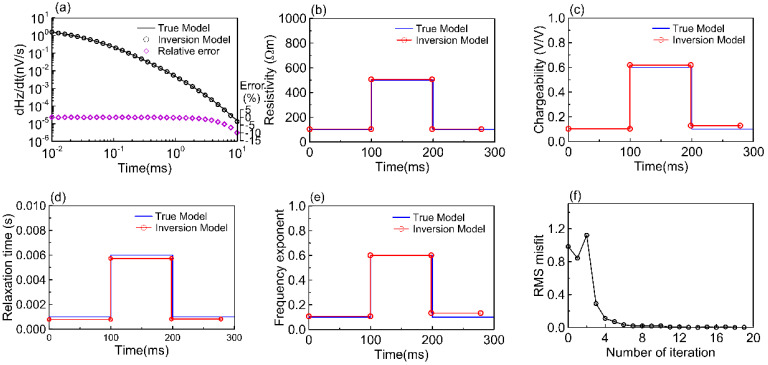
Model II inversion results of the Geotech waveform. (**a**) Data RMS misfit between true model modeling and inversion model simulation. Synthesized data (line) and forward response (circle mark); black (positive); relative error (purple diamond). (**b**–**e**) Represent resistivity, chargeability, relaxation time, frequency exponent, in that order. (**f**) RMS misfit for the number of iterations.

Figure 5 shows the inversion results of Model I. The first two layers of resistivity and frequency exponent inversion results are particularly consistent with the true model, while the chargeability of the third layer is lower than that of the true model. The first layer relaxation time is smaller than the true model, but the three-layer boundary depth of the relaxation time is consistent with the true model. Figure 6 shows the inversion results of Model II. The model layer thicknesses of resistivity, chargeability, relaxation time, and frequency exponent dovetail well with the four true models, and the four parameters are very close to the four true models. The data misfits (RMS) of the various models in Figure 5 and Figure 6 achieve rapid inversion within 14 iterations and converge to the expected error. The overall fit of the attenuation curve is good, with less than 1% relative error for each time channel, while the negative error of the attenuation curve is −8 to −23%, particularly the relative error between the current result of the inversion model and the synthetic data of the true model, as shown in Figure 5a, and only the last three time channels in Figure 6a have an error of more than 4%.

#### 2.5.2. Inversion in a Quasi-Two-Dimensional Space

To evaluate the effect of inversion using the four parameters of the Cole–Cole model, one-dimensional lateral constraint inversion (LCI) [23,24] was used to invert synthetic data from ATEM method investigations and Qingchengzi mine site data. The ATEM response can be simplified to the following equation:

(31) $$d_{obs}=F(P)$$

In the inversion, the observed data are the partial derivative of the magnetic field response to time. To obtain the observed data for a survey line with *N_s_* measuring points, the collected data are arranged in the form of column vectors $d_{obs}$:

(32) $$d_{obs}={\left[d_{obs1},d_{obs2},\ldots d_{obsNs}\right]}^{T}$$

where $d_{obsi}={\left[d_{obsi,1},d_{obsi,2}\ldots d_{obsi,TN}\right]}^{T}$, and TN is the time channel number of the *i*-th measuring point’s received data.

For the N-layer model, the model parameters of each measurement point are:

(33) $$p_{i}=\left[\rho_{i,1}\cdots \rho_{i,N},m_{i,1}\cdots m_{i,N},\tau_{i,1}\cdots \tau_{i,N},c_{i,1}\cdots c_{i,N},h_{i,1}\cdots h_{i,N-1}\right].$$

where *p_i_* is the model parameter of the first layer of the *i*-th measuring point and *h*_*i*,1_ is the thickness of the first layer of the *i*-th measuring point. The model parameter set is the thickness of the first layer of the first measurement point, and the model parameter set is:

(34) $$P={\left[p_{1},p_{2}\cdots p_{N_{s}}\right]}^{T}.$$

Perform a first-order Taylor expansion for *F* and ignore higher-order terms. Equation (31) can be obtained:

(35) $$\Delta d_{obs}=d_{obs}-F(P_{0})\approx J(P-P_{0})+e_{obs}$$

The main purpose of LCI inversion is to produce smoothness by minimizing the error of geoelectric characteristics between adjacent data locations. It constrains the minimization of thickness differences between IP parameters and adjacent measurement points by modifying the objective function. Lateral constraint inversion includes data fitting terms and model constraint terms.

(36) $$\left[\begin{array}{l} \Delta d_{obs}\\ \Delta r \end{array}\right]=\left[\begin{array}{l} W_{1}J\\ W_{2}R \end{array}\right]\Delta m+\left[\begin{array}{l} e_{obs}\\ e_{r} \end{array}\right].$$

where Δ*r* = *RP* and *R* is the lateral constraint operator, which is a sparse matrix composed of 1 and −1. Equation (36) is simplified to:

(37) $$\Delta d=J^{\prime }\Delta p+e^{\prime }$$

where *J*^′^ is the joint Jacobian matrix, the damped least squares method is used to solve Equation (37), and SVD is used to decompose the joint Jacobian matrix for inversion. The model updating equation is obtained as:

(38) $$\Delta p=V{\left(S^{2}+\lambda^{2}I\right)}^{-1}SU^{T}\Delta d$$

where *S* is the singular value matrix, *U* and *V* are the eigenvector matrices, and *λ* is the damping factor.

To select the optimal damping factor, the initial value of *λ* is set to the maximum of the diagonal elements of the singular value matrix Λ and then searched according to the method of decrement [25]. When the fitting difference is no longer part of the simplified equation, the current λ is the damping factor chosen for this iteration. Consider the nonlinearity of the actual inversion problem, starting with the selected initial model and Equation (38) for iterating until the error function is less than a preset threshold.

**Figure 7:**
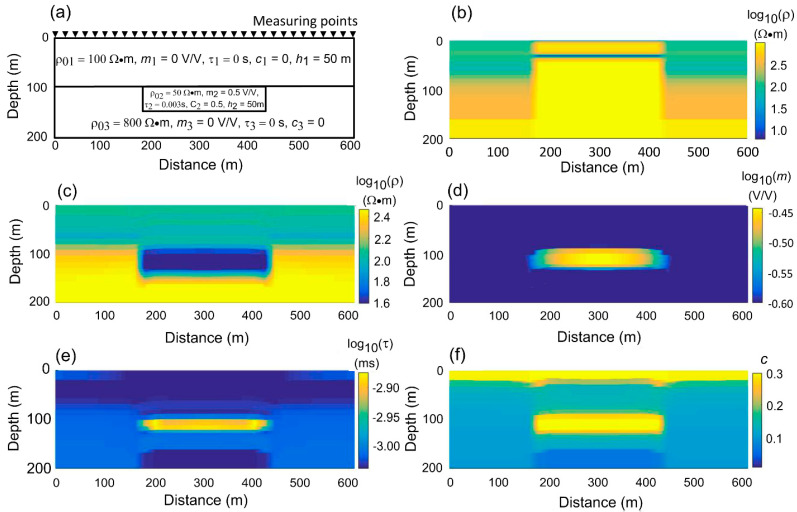
The results of inversion with four parameters of the Cole–Cole model or alone for resistivity. (**a**) Model III mesh has 50 m point spacing and 20 m layer thicknesses, and the transmitting and the receiving points are on the same point, for a total of 30 measuring points. (**b**) The resistivity section on its own for inversion of resistivity. (**c**–**f**) Sections for resistivity, chargeability, relaxation time, and frequency exponent inversion for four Cole–Cole model parameters.

**Figure 8:**
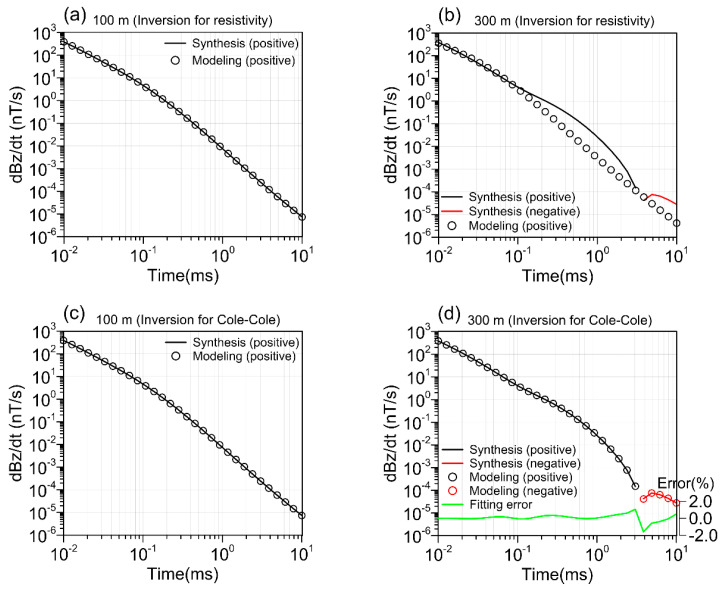
Two fitting points of inversion of the Cole–Cole model or alone for resistivity. (**a**) Inversion for resistivity at 100 m, (**b**) Inversion for resistivity at 300 m, (**c**) Inversion for Cole–Cole at 100 m, (**c**) Inversion for Cole–Cole at 300 m. Model synthesis data (line) and forward response (circle mark); black (positive) and red (negative); fitting error (green).

In the two-layer model (Model III), an LCI inversion of Cole–Cole IP parameters is performed using model data from a model with a low-resistivity, high-chargeability body in Figure 7a. As shown in Figure 7b, the model that only inverts resistivity is completely disconnected from the real model, and Figure 8a also shows that the sounding curve at 100m without chargeability in the model is well fitted, while the sounding data at 300m on the chargeability body fails to be fitted due to sign reversal (Figure 8b). Figure 7c,f shows the results of inverting the four parameters of the Cole–Cole model. Except for a layer of anomalies above the frequency exponent, the models of the other four parameters have recovered well. The fitting of the two curves in Figure 8c,d also shows that the sounding curves at 100 m and 300 m are well fitted, and the fitting error is less than 2%. The results show that in order to invert from IP data, Cole–Cole model parameters must be used to restore the true geoelectric structure.

### 2.6. Inversion Velocity Analysis

To calculate the data in this study, an I7-6700 CPU chip and a 16 GB memory PC were used. In this study, six tests on the inversion time of Jacobi’s analytical method and perturbation method were performed on the synthesized data of several models. The advantages of the analytical method in terms of operation speed are shown in Table 3. When the resistivity and thickness of the three-layer model are just 1-D inversion, the analytical method’s and perturbation method’s inversion times are 4.23 times and 2.49 times, respectively. The ratio is bigger than 2.24 in the one-dimensional inversion of parameters ρ, *m*, *τ*, *c*, and *h* of Model I and Model II of the three-layer model. However, for Model III with 10 layers and 30 sounding sites, the quasi-two-dimensional inversion time is extremely long, taking 35,188.167 s for the analytical technique and 259,200.457 s for the perturbation method, with a ratio of 7.37 times. This demonstrates that the analytical technique’s computational speed is faster than the perturbation method’s, particularly for the quasi-two-dimensional inversion with numerous layers and multiple parameters, where the analytical method considerably decreases the computation time.

## 3. Field Example

### 3.1. The Survey

**Figure 9:**
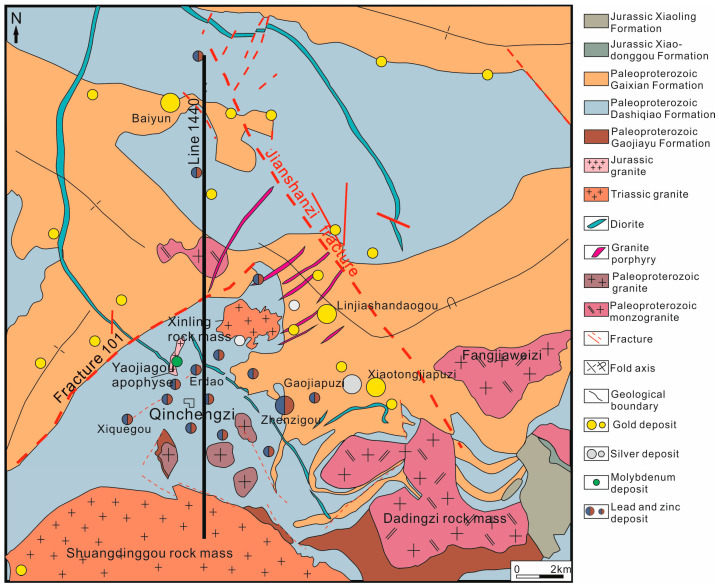
A geological map of the flight area. The 1440 line in this figure is an inversion profile.

The measured data come from an ATEM technology study conducted in the Qingchengzi Mining Area of China’s Liaodong Peninsula in the northeast of Craton. The geological conditions of the study area are superior, with many polymetallic deposits such as lead, zinc, gold, and silver located in the middle of the Liaodong section of the Liaoji Rift Valley. At one of the most important polymetallic deposits in China, proven reserves contain over 1.6 million tons of lead and zinc deposits, over 300 tons of gold, and over 4000 tons of silver [26]. Due to the long history of mining and the rapid depletion of resources, the mining area has become one of the nearly exhausted reserves mines in China. It is thought that there is a high probability of more mineralization being found in and around Qingchengzi. However, more than 80% of the area is mountainous, and more than 70% is forested, and the terrain is more complex (Figure 9). As a result, there has not been enough geological research and prospecting in the area. The survey was commissioned by the Institute of Geology and Geophysics of the Chinese Academy of Sciences. It covered 456 square kilometers in the Qingchengzi region.

Metamorphic rocks in the Qingchengzi region have high carbon and pyrite content [27], which will have a greater IP impact on this AEM study. In fact, a considerable number of IP effect characteristics are observed in the measured data, which requires IP model inversion to restore the accurate geoelectric structure and prevent false resistivity anomalies. Here is shown the inversion structure of the 18 km route profile through the Qingchengzi lead–zinc mine.

### 3.2. ATEM System

In the central loop configuration of the Geotech system used in this survey, the EM bird has an average flight altitude of 58.7 m, a flight speed of 80 km/h, a peak dipole moment of 400,000 NIA, and a trapezoidal pulse width of 7.385 ms, as shown in Figure 1a. Forty-eight time channels are used to capture data between 0.016 and 10.667 ms after the end of time. Table 4 shows the basic characteristics of the transmitter and receiver system.

### 3.3. Inversion Results

**Figure 10:**
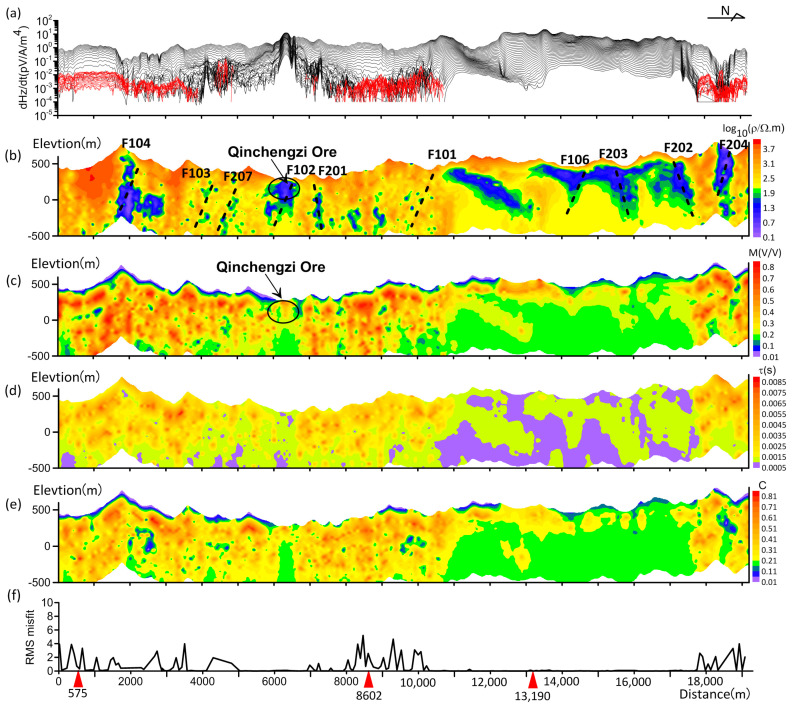
Line 1440 data and inversion result. (**a**) dHz/dt data normalized by transmit current, transmit coil area, and receive coil area, with positive and negative measured data values represented by black and red lines in the figure, respectively. (**b**–**e**) Inversion sections of the resistivity, chargeability, relaxation time, and frequency exponent. (**f**) RMS misfit section.

The Geotech company provided inversion data, and Geotech also performed coil non-horizontal correction and receiver filtering. We deleted poor-quality and selected low-noise data from the collected data from every four measurement points in a large number of data points without averaging smooth processing. The measurement data for selecting the line 1440 is shown in Figure 10a. Positive values are represented in black, while negative values for IP responses are represented in red. The initial inversion model has 11 layers. Each layer has the same initial parameters (ρ_0_ = 100 Ω·m, *m* = 0.1, *τ* = 0.001 s, *c* = 0.3, *h* = 50 m), a maximum number of iterations of 15, and a termination error of 0.0001. Figure 10b–e shows the inversion results for the resistivity, chargeability, relaxation time, and frequency exponent parameters, and Figure 10f shows the data misfit (RMS).

**Figure 11:**
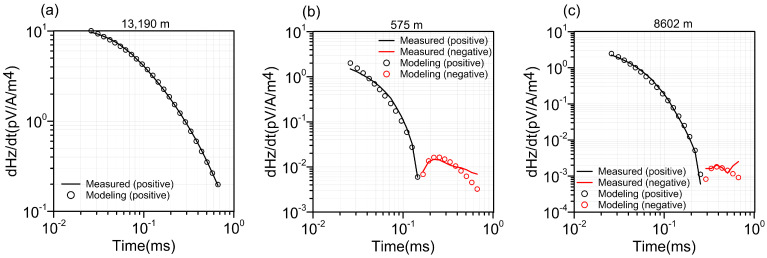
Three fitting examples of inversion data sounding points. (**a**) 13,190 m, (**b**) 575 m, (**c**) 8602 m. Measured data (line) and forward response (circle mark); black (positive) and red (negative).

Figure 11 shows the fitting of the simulated data of the three typical sounding points. When the measured data in Figure 11a have no negative number, the sounding curve fits well, while when the two sounding datasets in Figure 11b,c have sign reversal, the two sounding curves do not fit well. The measured curves in Figure 10a have negative response represented by red lines, and the simulated response of the inversion model is highly consistent with the measurement data. The measured curves in Figure 10b,c both have negative responses, and the model response is about the same as the pattern of the measured data but does not achieve a high fit, which is why the RMS corresponding to the negative value area in Figure 10a is larger. It has little effect on the negative response contour fit inversion, as shown in Figure 9b–e. The resistivity profile of Figure 10b clearly shows 10 low-resistivity anomalies corresponding to the true fault. According to Figure 10c–e, most of the high chargeability, relaxation time, and frequency exponent indicate high-chargeability anomalies, such as carbonaceous rocks, in different formations, respectively. The Qingchengzi lead and zinc deposits had low resistivity and high chargeability between 5900 and 6800 m, and the low resistivity and high chargeability related to the gold deposits were found between 13,100 and 19,000 m. At 11,000–13,000 m, there is a northward tilt of low-resistivity and high-chargeability anomalies, which may be related to the containing orebody. The measured data inversion results show that LCI can invert the four parameters of Cole–Cole and can invert the orebody or carbon formation form and two-dimensional underground structure.

## 4. Conclusions

Based on the Cole–Cole model and the transmitter waveform, we propose a Jacobian matrix chain analysis formula and invert the zero-frequency resistivity, chargeability, relaxation time, and frequency exponent using the least squares method. Here are the main conclusions.

Numerical simulation results show that the analytical formula can significantly shorten the inversion calculation time. Simulations of the waveform response of Geotech systems were shown to be accurate by comparing simulations of the waveform response of the Geotech system with simulations of four different transmitter waveforms (rectangular, trapezoidal, semi-sinusoidal, and Geotech). The accuracy of the Jacobian matrix calculation is confirmed by comparing the Jacobian matrix based on the transmitter current waveform with the perturbation differential method.

In the inversion of synthetic data, the inversion of the resistivity parameter alone does not replace the inversion of the four Cole–Cole parameters. The four parameters of the Cole–Cole model are inverted in synthetic data, and each parameter and resistivity value for each layer depth are close to the true model.

LCI can be used to reverse the 2-D model structure as much as possible, although 1-D inversion has limitations on 2-D or 3-D structures. The inversion results of the field data show that there are many measurement points in the measured profile with negative attenuation. However, through the inversion of IP parameters, a reasonable resistivity structure and a satisfactory match can be obtained.

The IP effect may lead to the symbolic inversion of the attenuation curve. The inversion in this paper does not make logarithmic calculations for the observational data. When the value range of the attenuation curve is wide, it leads to poor fitting of the late channel data, especially the negative value of the IP effect, which affects the true reflection of deep geological information or ore bodies. Therefore, further research is needed on IP inversion.

## Figures and Tables

**Table 1:** Cole–Cole parameter ranges from several deposits [22].

	ρ_0_ (Ω·m)	*M* (V/V)	*τ* (s)	*c*
Porphyry (dry)	1 × 10^1^–1 × 10^3^	0.1–0.5	1 × 10^−5^–1 × 10^0^	0.1–0.6
Porphyry (wet)	1 × 10^0^–1 × 10^4^	0.1–0.8	1 × 10^−2^–7 × 10^4^	0.1–0.5
Magnetite	1 × 10^1^–1 × 10^3^	0.1–1.0	8 × 10^−4^–3 × 10^0^	0.1–0.6
Pyrrhotite	1 × 10^0^–1 × 10^3^	0.3–0.8	3 × 10^0^–1 × 10^5^	0.1–0.5
Massive Sulfide	1 × 10^−2^–1 × 10^3^	0.6–0.95	8 × 10^−4^–2 × 10^0^	0.1–0.4
Graphite	1 × 10^−2^–1 × 10^3^	0.75–0.98	8 × 10^1^–8 × 10^3^	0.1–0.5

**Table 2:** Errors in the analytic and perturbation methods.

	Analytical Method				Perturbation Method				Error (%)			
Time (ms)	*ρ*	*m*	*τ*	c	*ρ*	*m*	*τ*	c	*ρ*	*m*	*τ*	c
0.010	1.056 × 10^−8^	5.260 × 10^−7^	7.302 × 10^−6^	1.844 × 10^−7^	1.050 × 10^−8^	5.282 × 10^−7^	7.230 × 10^−6^	1.827 × 10^−7^	0.57	−0.42	1.00	0.93
0.020	1.568 × 10^−4^	7.150 × 10^−3^	0.1464	3.016 × 10^−3^	1.559 × 10^−4^	7.180 × 10^−3^	0.1455	2.999 × 10^−3^	0.58	−0.42	0.62	0.57
0.042	6.333 × 10^−3^	0.2512	7.742	0.1184	6.289 × 10^−3^	0.2524	7.698	0.1181	0.70	−0.48	0.57	0.25
0.085	1.178 × 10^−2^	0.3630	18.12	0.1594	1.168 × 10^−2^	0.3646	18.02	0.1595	0.86	−0.44	0.55	−0.06
0.174	8.353 × 10^−4^	4.070 × 10^−2^	2.499	5.558 × 10^−2^	8.291 × 10^−4^	4.103 × 10^−2^	2.492	5.546 × 10^−2^	0.75	−0.8	0.28	0.22
0.356	2.176 × 10^−3^	7.288 × 10^−2^	3.604	3.932 × 10^−2^	2.157 × 10^−3^	7.322 × 10^−2^	3.585	3.938 × 10^−2^	0.88	−0.46	0.53	−0.15
0.728	6.711 × 10^−4^	1.300 × 10^−2^	1.176	3.262 × 10^−4^	6.643 × 10^−4^	1.303 × 10^−2^	1.171	3.266 × 10^−4^	1.02	−0.23	0.43	−0.12
1.487	9.243 × 10^−5^	3.142 × 10^−4^	0.1374	2.064 × 10^−3^	9.142 × 10^−5^	3.091 × 10^−4^	0.1369	2.069 × 10^−3^	1.10	1.65	0.37	−0.24
3.039	7.012 × 10^−6^	2.353 × 10^−4^	7.205 × 10^−4^	5.165 × 10^−4^	6.930 × 10^−6^	2.371 × 10^−4^	7.285 × 10^−4^	5.169 × 10^−4^	1.18	−0.76	−1.10	−0.08
6.210	8.104 × 10^−8^	6.396 × 10^−5^	2.969 × 10^−3^	8.262 × 10^−5^	8.024 × 10^−8^	6.426 × 10^−5^	2.952 × 10^−3^	8.245 × 10^−5^	1.00	−0.47	0.58	0.21
10.00	1.136 × 10^−7^	2.212 × 10^−5^	1.368 × 10^−3^	2.202 × 10^−5^	1.126 × 10^−7^	2.220 × 10^−5^	1.361 × 10^−3^	2.190 × 10^−5^	0.89	−0.36	0.51	0.55

**Table 3:** Comparison of inversion time length between perturbation and interpretation method.

Test Name	Model Parameters	Parameters of Inversion	Number of Iterations	Duration of Perturbation Method	Duration of Analytical Method	Ratio of Perturbation to Analytical
Test 1	ρ_0_ = {200, 50, 300} Ω·m *h* = {50, 50} m	ρ and *h*	6	149.063	35.255	4.23
Test 2	ρ_0_ = {200, 50, 500} Ω·m *h* = {50, 50} m	ρ and *h*	20	2101.168	842.649	2.49
Test 3	ρ_0_ = {100, 500,100} Ω·m *h* = {50, 50} m	ρ and *h*	20	2001.307	798.386	2.51
Test 4	Model I (3 layers)	ρ, *m*, *τ*, *c*, and *h*	15	2253.226	685.001	3.29
Test 5	Model II (3 layers)	ρ, *m*, *τ*, *c*, and *h*	20	2129.826	950.407	2.24
Test 6	Model III (10 layers)	ρ, *m*, *τ*, *c*, and *h*	15	259,200.457	35,188.167	7.37

**Table 4:** Geotech system parameters used in the Qingchenzi survey.

Transmitter		Receiver	
Transmitter loop diameter	26 m	Receiver loop diameter	1.2 m
Number of transmitter loop turns	4	Number of receiver loop turns	100
Transmitter area	2132.7 m^2^	Receiver area	113.04 m^2^
Basic frequency	25 Hz	Sampling	0.2 s, positioning accuracy: 1.2 m
Peak current	Max. 310 A usual: 200 A		
Pulse width	7.385 ms		
Waveform	Bipolar trapezoidal wave		
Peak moment	400,000 NIA		

## Data Availability

Data associated with this research are available and can be obtained by contacting the corresponding author.
